# A nurse-delivered mental health intervention for obstetric fistula patients in Tanzania: results of a pilot randomized controlled trial

**DOI:** 10.1186/s40814-017-0178-z

**Published:** 2017-09-12

**Authors:** Melissa H. Watt, Mary V. Mosha, Alyssa C. Platt, Kathleen J. Sikkema, Sarah M. Wilson, Elizabeth L. Turner, Gileard G. Masenga

**Affiliations:** 1 Duke Global Health Institute, Duke University, Duke Box 90519, Durham, NC 27708 USA; 2 Kilimanjaro Christian Medical Centre, Moshi, Tanzania; 3 Department of Biostatistics and Informatics, Duke Global Health Institute, Duke University, Durham, NC USA; 4 Department of Psychology and Neuroscience, Duke Global Health Institute, Duke University, Durham, NC USA

**Keywords:** Tanzania, Obstetric fistula, Mental health, Intervention, Pilot

## Abstract

**Background:**

Obstetric fistula has severe psychological consequences, but no evidence-based interventions exist to improve mental health in this population. This pilot trial evaluated a psychological intervention for women receiving surgical care for obstetric fistula.

**Methods:**

A parallel two-armed pilot RCT was conducted between 2014 and 2016. The intervention was six individual sessions, based on psychological theory and delivered by a nurse facilitator. The study was conducted at a tertiary hospital in Moshi, Tanzania. Women were eligible if they were over age 18 and admitted to the hospital for surgical repair of an obstetric fistula. Sixty participants were randomized to the intervention or standard of care. Surveys were completed at baseline, post-treatment (before discharge), and 3 months following discharge. Standardized scales measured depression, anxiety, traumatic stress, and self-esteem. Feasibility of an RCT was assessed by participation and retention. Feasibility and acceptability of the intervention were assessed by fidelity, attendance, and participant ratings. Potential efficacy was assessed by exploratory linear regression and clinical significance analysis.

**Results:**

Eighty-five percent met criteria for mental health dysfunction at enrollment. All eligible patients enrolled, with retention 100% post and 73% at 3 months. Participants rated the intervention acceptable and beneficial. There were sharp and meaningful improvements in mental health outcomes over time, with no evidence of differences by condition.

**Conclusions:**

A nurse-delivered mental health intervention was feasible to implement as part of in-patient clinical care and regarded positively. Mental health treatment in this population is warranted given high level of distress at presentation to care.

**Trial registration:**

ClinicalTrials.Gov NCT01934075.

## Background

Obstetric fistula is a devastating result of childbirth, borne out of poverty, poor access to maternal health care, and gender inequities [1–3]. Women develop obstetric fistula due to prolonged obstructed labor, or when a perforation is made during a cesarean section. The result is a hole between the bladder or rectum and the vagina, which causes uncontrollable leaking of urine and/or feces. The condition is nearly non-existent in high-income countries where emergency obstetric care is widely available [4]. Although the exact prevalence of obstetric fistula is unknown, it is estimated that over 1–2 million women are living with a fistula globally [5, 6].

The mental health impact of obstetric fistula can be devastating, due to both the traumatic childbirth and the resulting physical condition [1, 7]. Women who develop a fistula typically experience several days of painful labor, usually ending in stillbirth, and physical complications such as nerve damage, infections, and infertility [8, 9]. These traumas are compounded by the humiliating condition of incontinence. Women with obstetric fistula report high levels of social isolation [10], divorce [11], stigma [12, 13], depression [14, 15], and general mental health dysfunction [7, 16, 17].

Surgical repair may heal or improve an obstetric fistula, and many countries such as Tanzania have established free fistula repair programs [18]. Women who are admitted for repair may spend up to 1 month or more in the hospital, providing a window of opportunity to address the psychological symptoms accumulated from living with this socially marginalizing condition and to develop coping skills to facilitate reintegration after repair. Although the need to address mental health issues in this population has been recognized [19–21] and is part of the WHO’s guiding principles of fistula management [22], to date no intervention studies have evaluated empirically supported psychotherapies to assist in emotional healing among fistula patients.

The objective of this study was threefold: (1) to assess the feasibility of conducting a full-scale randomized controlled trial (RCT) of a mental health intervention with obstetric fistula patients; (2) to determine the feasibility and acceptability of a six-session, nurse-delivered mental health intervention for obstetric fistula patients; and (3) to consider the potential efficacy of the intervention on mental health outcomes.

## Methods

### Study design

A pilot RCT was conducted between March 2014 and June 2016 [23, 24]. A target sample size of 60 was chosen as adequate to examine the feasibility and acceptability of a pilot RCT with two conditions [25]. We estimated the power to detect a difference in 3-month primary outcomes for the sample size of 60 women (30 per group) would be 77%, based on ANCOVA and assuming within-person correlation between baseline and 3-month depression scores of 0.7 and a pooled standard deviation (SD) of 12. Such a scenario would correspond to an effect size of 0.5.

Reporting of study methods and results followed the CONSORT 2010 statement, as extended to pilot RCT trials [26]. A full study protocol is available from the corresponding author.

### Setting

All recruitment and procedures took place at Kilimanjaro Christian Medical Centre (KCMC) in Moshi, Tanzania. KCMC has a dedicated 12-bed fistula ward and conducts approximately 40 fistula repair surgeries per year. Patients admitted for surgical repair of a fistula undergo surgery normally within 1 week after admittance and remain in the ward for 2–3 weeks after surgery. During the course of the study, there were anywhere from zero to five patients admitted for fistula repair surgery any one time. Surgeries are conducted free of charge to patients, supported by a national fistula repair initiative [27].

### Participants

Women were eligible to participate in the study if they were 18 years or older and admitted to KCMC for surgical repair of a fistula that was a result of childbirth, either due to obstructed labor or iatrogenic causes during a cesarean section. Women were eligible regardless of time living with fistula and any previous repairs. Women were ineligible if they did not speak Swahili or could not provide informed consent due to mental capacity or severe illness.

### Procedures

Women admitted to KCMC for a suspected obstetric fistula were examined by the lead fistula surgeon at KCMC, with a dye test used to confirm the presence or absence of a fistula. Women with a confirmed fistula were referred to the study coordinator, who used an unstructured clinical interview to assess eligibility criteria. Eligible women were told about the study and invited to participate.

After providing consent, women completed the baseline assessment and were randomized to one of two conditions: standard of care control or psychotherapy intervention. A random number generator was used to create the randomization sequence. Randomization was conducted in blocks of ten, in order to ensure balanced assignment over time. Condition assignments were placed in sealed envelopes, blinded to the local study team until assignment. Due to the nature of the intervention, neither women nor study staff could be blinded to condition assignment. The statistician who conducted the data analysis was blinded to intervention condition, with intervention condition revealed at the conclusion of analysis. Women randomized to the control condition received the standard of care, which included social support by hospital nursing staff in the context of clinical care, but no formalized counseling. Women randomized to the intervention condition received six individual treatment sessions during their hospital stay, in addition to all standards of care services at the hospital. The post assessment was conducted within 48 h prior to discharge from the hospital (on average 3 weeks after baseline). The follow-up assessment was conducted at approximately 3 months after discharge. Study participants provided multiple forms of tracking information at discharge, including cellphone numbers for themselves (if available) and for their friends/relatives. Prior to the participant’s follow-up date, the study coordinator helped the woman to plan her return visit to the hospital and transferred funds for her bus fare via cellphone (a common payment method in Tanzania).

Incentives for participation varied by assessment time-point. Upon enrolment, participants received a gift bag with toiletries; at the post assessment, they received a *khanga* (local cloth); and at the 3-month follow-up they received TSh 20,000 (approximately ten US dollars), in addition to reimbursement for all relevant travel and lodging expenses.

### Intervention

*Uponyaji* (the Swahili word for *heal)* was a six-session, individual psychological intervention. The manualized curriculum was developed through extensive qualitative work with a range of stakeholders, and based on theories of cognitive behavioral therapy [28] and coping models [29]. By helping patients to reframe their experience with a fistula and develop coping skills to deal with the physical and social impact of the fistula, the intervention aimed to reduce patient distress, improve self-esteem and optimism, and therefore support successful community reintegration following repair. The intervention sessions were approximately 60 min long and delivered by a single intervention facilitator, a community health nurse who received training by the study principal investigator and clinical psychologists. Participants in the intervention condition received two sessions prior to surgery (content focused on setting personal goals for treatment and creating a new story about the fistula based on clinical knowledge), and four sessions following surgery during post-operative recovery (content focused on psychoeducation related to thoughts and emotions; strategies for coping; social relationships; and planning for the future). The intervention content has been described in full elsewhere [30].

### Survey measures

The surveys at each time-point included ~250 questions and took ~90 min to complete. Selection of measures was informed by the team’s previous work with this population [17, 31]. Measures were translated into Swahili and independently back-translated into English, with discrepancies resolved by a third party. The survey was orally administered. For Likert-scaled measures, an aid was used to visually display response options, and participants could either verbally provide a response or point to the option [32].

#### Demographics and obstetric history

Patients provided demographic information (age, education, ability to read, employment, religion, relationship status), and self-reported information about their obstetric history (fistula type, stillbirths related to fistula, time living with fistula, number of previous fistula surgeries, other living children).

#### Depression

The Centre for Epidemiologic Studies Depression Scale (CES-D) [33] assesses depressive symptoms in the past week on a 4-point Likert scale. The 20-item measure yields a depression severity score ranging from 0 to 60 (*α* = 0.89). Moderate to severe depression symptomatology was characterized by a score ≥ 16 [33].

#### Generalized anxiety

The Beck Anxiety Inventory (BAI) assesses the presence of common physical, psychological, or cognitive symptoms of anxiety during the past week on a 4-point Likert scale [34–36]. The 21-item measure yields a score ranging from 0 to 63 (*α* = 0.90). Moderate to severe anxiety symptomatology was characterized by a score ≥ 19 [34–36].

#### Traumatic stress

The PTSD Checklist-Civilian Version (PCL-C) [37] assesses traumatic symptoms, irrespective of a specific trauma. Questions are specific to the past month, and responses are measured on a 5-point Likert scale. The measure yields a traumatic stress score ranging from 17 to 85 (*α* = 0.86). Scores ≥ 30 were considered as severe symptomatology, indicative of post-traumatic stress disorder (PTSD) [37].

#### Self-esteem

The Rosenberg Self-Esteem Scale assesses participants’ global self-worth by assessing both positive (e.g., “On the whole, I am satisfied with myself”) and negative (e.g., “At times, I think I am no good at all”) feelings about the self [38]. Items were slightly modified for use with a fistula population [39]. Participants are asked how much they agree with each item on a 4-point Likert scale. The 10-item measure yields a self-esteem score ranging from 0 to 30 (*α* = 0.79).

#### Surgical success

Prior to discharge, the lead fistula surgeon at KCMC conducted a clinical exam, noting the degree of residual incontinence and the likelihood that the participant would need a repeat surgery. The surgery was considered unsuccessful if the participant had anything more severe than stress incontinence and if she was assessed as having an incurable fistula or needing an additional surgery.

### Indicators of trial feasibility

Trial feasibility was assessed using multiple data points that are considered best practice for assessing feasibility in a pilot RCT [24]. We recorded the proportion of eligible women who agreed to participate in the study, with a 70% uptake being our benchmark for feasibility, and aimed to recruit an average of four patients per month. We assessed whether the research team could effectively randomize participants into conditions, and whether participants accepted their assigned condition. We then considered the proportion of enrolled participants who completed each assessment time-point (baseline, post, 3-month), with a 60% retention rate at each time being our benchmark for feasibility.

### Indicators of intervention feasibility and acceptability

Intervention feasibility was assessed by the proportion of intervention sessions attended (benchmark of 80%) and fidelity to each session, which were recorded by the intervention facilitator. After each session, the interventionist wrote detailed notes using a semi-structured format to record her experience delivering the session. Regular debriefing meetings were held as a team to discuss the process of intervention implementation.

Acceptability of the intervention was assessed from participants in the intervention condition with 15 questions at the post-treatment survey. We asked five questions about satisfaction with various domains, each with structured response options (“Would you take part in this study again?”, “How satisfied were you with the intervention?”, “How satisfied were you with the intervention therapist?”, “What did you think about the number of sessions?”, and “What did you think about the time spent at each session?”). We also asked four open-ended questions, covering details about what the participant liked, disliked, would what want to see changed, and any general feedback on the overall structure and content of the intervention. Finally, we asked a question about the perceived usefulness of each of the six sessions (responses ranging from not useful to very useful).

### Data analysis to evaluate potential intervention efficacy

#### Regression analysis

The primary endpoints for efficacy of the intervention were symptomatology of depression, anxiety and PTSD, as well as self-esteem. The size of the pilot study (*N* = 60) did not allow for adequately-powered hypothesis testing of treatment efficacy. Instead, an exploratory regression analysis was conducted using mixed effects linear regression with individual-level random intercepts. All assessments (baseline, post, and follow-up) were combined in a longitudinal linear mixed effects regression using a traditional time by condition modeling strategy [40], with binary indicators for treatment condition, time, and treatment by time interactions. Regressions were adjusted by length of time with fistula (years, mean-centered and divided by standard deviation), and exploratory efficacy was presented as mean estimates of differences between arms in change scores with 95% confidence intervals.

#### Clinical significance analysis

We conducted a secondary exploratory analysis on subsets of women meeting symptomatology criteria that are indicative of diagnoses for depression, anxiety, and PTSD. Clinical significance methods were used to look for evidence of possible moderating effects of success of surgery on intervention outcomes [41]. A cut-off score was computed to determine whether an individual’s post-treatment score was more likely to be drawn from a “functional” vs. a “dysfunctional” population. Dysfunctional distributions were defined using baseline depression, anxiety, and PTSD scores for study participants meeting criteria for depression, anxiety, and PTSD, respectively. Functional distributions for depression and PTSD were defined using pre-existing data (*n* = 65 and *n* = 49, respectively) collected in a general gynecological population from KCMC in Tanzania [17]. Functional distributions of anxiety scores were not available from pre-existing data, thus we used baseline scores from our own sample of women that did not meet the criteria for anxiety (*n* = 35). Next, a reliable change index (RCI) was calculated for each of the three measures by dividing each individual’s change scores by the standard error of measurement for the functional population, correcting for measurement error. An RCI less than or equal to −1.96 was classified as “reliably improved,” an RCI of more than 1.96 was classified “reliably deteriorated,” and an RCI with an absolute value less than 1.96 represented “no change.” [42] In order to be considered “recovered”, an individual’s follow-up score had to be below the cut-off point, and that change had to be classified as “reliably improved.” We present classifications of the change scores based on the RCI in addition to the count of those who “recovered,” separately by whether the participant had a successful surgical outcome and by treatment condition.

## Results

### Trial feasibility

**Fig. 1:**
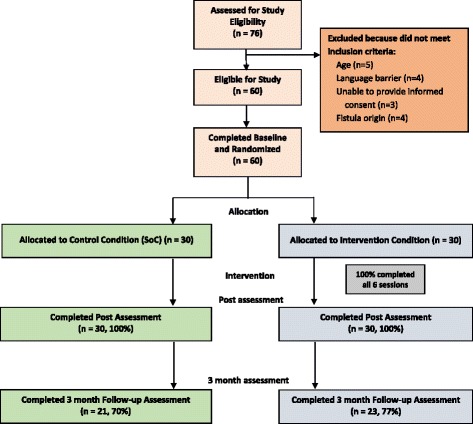
Flow of the study participants through the trial

Over the study period, all fistula patients admitted to KCMC (*n* = 76) were assessed for their eligibility for the study (Fig. 1). Sixteen women were excluded from participation because they were under age 18 (*n* = 5), did not speak Swahili (*n* = 4), could not provide informed consent due to serious co-morbidities or diminished mental capacity (*n* = 3), or had a fistula that was due to non-obstetric causes (e.g., sexual trauma or hysterectomy; *n* = 4). A total of 60 women were eligible for study participation, and all provided informed consent to enroll. Enrolment was slower than expected, at a rate of approximately 2.5 per month.

All study participants completed the baseline and the post-treatment assessment (*n* = 60; 100%). Forty-four participants (73.3%) returned for the 3-month assessment, with slightly higher follow-up in the intervention condition (76.7 vs. 70%). Median travel time was 9 h (range: half hour to 12 h, SD = 4 h) from their homes to KCMC for the return visit. Of the 16 who did not return, 10 could not be reached by telephone, 5 were reached but declined to come, and one had a follow-up date that fell outside of the funded study period. Reasons given for not returning included being too ill or frail to travel, needing to stay home to care for a sick relative, and being disappointed that their surgery was not successful. Women were less likely to return for the 3-month assessment if at post they had elevated anxiety (0% for those who returned vs. 19% for those who did not return) or PTSD (39 vs. 63%), or if their surgeries were unsuccessful (25 vs 56%).

The baseline sociodemographic characteristics of the sample are described in Table 1. Overall, women had a median age of 39.5 years, had low levels of education (only 52% completed primary school), had experienced stillbirth due to fistula (90%), and had living children (67%). At baseline, women in both arms presented with high levels of distress, with 51 women (85%) meeting criteria that were indicative of diagnoses for at least one type of mental health disorder (67% for depression; 42% for anxiety; 73% for PTSD). Despite the small sample size, study arms were reasonably balanced between conditions in both their sociodemographic characteristics (Table 1) and their mental health distress at baseline (Table 2).

**Table 1:** Description of pilot trial participants at baseline (*n* = 60) and of surgical outcome, *n* (%) unless otherwise stated

	Control (*N* = 30)	Intervention (*N* = 30)	Total (*N* = 60)
Age, median (IQR)	37.0 (25, 55)	40.5 (27, 54)	39.5 (26.5, 54.5)
Age			
18 to 24	7 (24%)	5 (17%)	12 (20%)
25 to 34	6 (21%)	5 (17%)	11 (19%)
35 to 44	5 (17%)	8 (27%)	13 (22%)
45 to 60	6 (21%)	8 (27%)	14 (24%)
61 to 80	5 (17%)	4 (13%)	9 (15%)
Highest level of education completed			
None	8 (27%)	10 (33%)	18 (30%)
Less than standard 7	7 (23%)	4 (13%)	11 (18%)
Completed standard 7	15 (50%)	16 (53%)	31 (52%)
Distance from home to KCMC (h), median (IQR)	9.3 (5, 11)	9.0 (2, 11)	9.0 (3, 11)
Literate	14 (47%)	17 (57%)	31 (52%)
Has any income-generating activities	20 (67%)	17 (57%)	37 (62%)
Religion			
Christian	13 (43%)	12 (40%)	25 (42%)
Muslim	17 (57%)	17 (57%)	34 (57%)
No religion	0 (0%)	1 (3%)	1 (2%)
Marital Status			
Married and living together	11 (38%)	13 (43%)	24 (41%)
Married but living apart	5 (17%)	2 (7%)	7 (12%)
Single (never married)	3 (10%)	3 (10%)	6 (10%)
Widowed	7 (24%)	5 (17%)	12 (20%)
Separated/divorced	3 (10%)	7 (23%)	10 (17%)
Experienced stillbirth/death due to fistula	28 (93%)	26 (87%)	54 (90%)
Has any living children	18 (60%)	22 (73%)	40 (67%)
Fistula type			
VVF^a^ (leaking urine)	26 (87%)	28 (93%)	54 (90%)
RVF^b^ (leaking stool)	1 (3%)	1 (3%)	2 (3%)
Both VVF and RVF	3 (10%)	1 (3%)	4 (7%)
Months from fistula, median (IQR)	154 (3, 295)	168 (10, 229)	162 (9, 295)
Fistula surgery successful^c^	18 (60%)	22 (73%)	40 (67%)

^a^Vesicovaginal fistula, ^b^Rectovaginal fistula, ^c^Surgical success was assessed by physician and defined as a closed fistula with no more than stress incontinence and no need for repeat surgery

**Table 2:** Psychological symptoms by time point and mixed effects linear regression mean estimates by time point and outcome (*N* = 60)

	Depression^a^		Anxiety^b^		PTSD symptoms^c^		Self-esteem^d^
Time-point	Observed values		Observed values		Observed values		Observed values
	Total score	Met criteria^a^	Total score	Met criteria^b^	Total score	Met criteria^c^	Total score
	Mean (SD)	*n* (%)	Mean (SD)	*n* (%)	Mean (SD)	*n* (%)	Mean (SD)
Baseline							
*Control* (*N* = 30)	25.9 (13.5)	21 (70.0%)	10.8 (9.6)	14 (46.7%)	38.8 (14.4)	21 (70.0%)	12.4 (6.8)
*Intervention* (*N* = 30)	22.1 (13.6)	19 (63.3%)	10.6 (10.5)	11 (36.7%)	37.1 (12.9)	23 (76.7%)	13.9 (7.2)
Post							
*Control* (*N* = 30)	12.4 (8.0)	10 (33.3%)	2.6 (5.8)	2 (6.7%)	31.1 (11.5)	15 (50.0%)	21.9 (6.0)
*Intervention* (*N* = 30)	9.9 (10.7)	5 (16.7%)	2.4 (3.3)	1 (3.3%)	28.6 (9.4)	12 (40.0%)	23.6 (4.5)
3-month follow-up							
*Control* (*N* = 21)	6.6 (9.5)	2 (9.5%)	1.0 (3.0)	1 (4.8%)	23.8 (7.3)	4 (19.0%)	24.0 (5.8)
*Intervention* (*N* = 23)	6.4 (7.0)	2 (8.7%)	2.1 (4.6)	1 (4.3%)	26.2 (7.8)	6 (26.1%)	25.0 (3.1)
Regression Estimates^e^							
Time period	Depression Mean Est. (95% CI)		Anxiety Mean Est. (95% CI)		PTSD symptoms Mean Est. (95% CI)		Self-esteem Mean Est. (95% CI)
Changes from baseline to post							
*Control*	−13.6 (−18.4,-8.7)		−8.2 (−11.3,-5.1)		−7.7 (−12.7,-2.7)		9.5 (6.9, 12.2)
*Intervention*	−11.7 (−16.6,-6.8)		−8.2 (−11.3,-5.1)		−8.3 (−13.4,-3.2)		9.8 (7.1, 12.4)
*Intervention* vs. *control*	1.9 (−5.0, 8.8)		−0.0 (−4.4, 4.4)		−0.6 (−7.7, 6.5)		0.2 (−3.5, 4.0)
Changes from baseline to 3-month follow-up							
*Control*	−18.8 (−24.3,-13.4)		−9.5 (−12.9,-6.0)		−14.7 (−20.3,-9.1)		11.6 (8.7, 14.6)
*Intervention*	−15.2 (−20.6,-9.8)		−8.6 (−12.1,-5.2)		−11.1 (−16.6,-5.6)		11.0 (8.1, 13.9)
*Intervention* vs. *control*	3.6 (−4.0, 11.3)		0.8 (−4.0, 5.7)		3.6 (−4.2, 11.5)		−0.6 (−4.7, 3.6)

^a^Depression measured by Center for Epidemiologic Studies Depression Scale (CES-D), criteria for depression = CES-D score at or above 16 (total range 0–60)

^b^Anxiety measure by Beck Anxiety Inventory (BAI), criteria for anxiety = BAI score at or above 10 (total range 0–63)

^c^PTSD Symptoms measure by PTS Checklist, criteria for PTSD = PTS Checklist score at or above 30 (total range: 17–85)

^d^Self-esteem measured by Rosenberg Self-Esteem scale (total range: 0–30)

^e^Regressions are linear mixed effects models with individual-level random intercepts. Estimates are presented as changes from baseline for each group with 95% confidence intervals. All estimates adjusted for length of time with fistula

### Feasibility and acceptability of the intervention

All women in the intervention condition received six intervention sessions as planned (two pre-surgery and four post-surgery). Intervention fidelity was high, with 96% of intervention content reported as fully or partially completed in the sessions. The clinical notes and debriefing meetings revealed that, overall, the patients connected well with the interventionist, and the intervention content allowed them to forthright in sharing their life experiences, emotions, and concerns. Many participants noted that the intervention provided them with a first opportunity to discuss their experience developing and living with a fistula, and many women noted that they had learned about female anatomy and the biological processes of birth for the first time. The intervention facilitator reported that patients grasped most intervention concepts easily; however, some women had difficulty with certain cognitive-behavioral skills such as distinguishing thoughts from emotions or choosing appropriate coping strategies.

In the post-treatment survey, satisfaction with the intervention was high. All participants in the intervention condition (30/30) stated that they would take part in the intervention again. Almost all (96.7%, 29/30) said that they were very satisfied or satisfied with the intervention, and 100% said they were very satisfied or satisfied with the intervention facilitator. While the majority of participants reported that they found both the number of sessions and time spent at the sessions to be the right amount, some found six sessions to be too many (20.0%, 6/30), and the time at each session to be too long (23.3%, 7/30). Participants indicated that they appreciated several aspects of the intervention, including gaining knowledge about how a fistula develops and the surgical process, discussing strategies for self-care, learning relaxation exercises, setting goals for the future, and receiving reassurance from the intervention nurse.

### Potential intervention efficacy

Unadjusted means for our outcomes of depression, anxiety, PTSD, and self-esteem were relatively balanced between the intervention and control arms at baseline (Table 2). Proportions of women meeting criteria indicative of diagnoses at baseline varied between arms, although differences in proportions were often based on small absolute numbers of women. Preliminary regression estimates indicate that, in both conditions, there were large and significant improvements in all mental health outcomes from baseline to post, and improvements that were as large or larger in magnitude from baseline to 3-month follow-up. The difference between conditions in the change in depression, anxiety, PTSD, and self-esteem was small in magnitude, with wide confidence intervals that were relatively symmetric around zero.

In the test of clinical significance, limiting the analysis sample for each outcome to only those women who met the criteria for mental health dysfunction at baseline for each of the outcomes resulted in 40 participants for depression (*n* = 14 with unsuccessful surgery), 25 participants for anxiety (*n* = 9 with unsuccessful surgery), and 44 participants for PTSD (*n* = 17 with unsuccessful surgery). *t* test and Cohen’s *D* tests confirmed that functional and dysfunctional distributions were statistically distinct from one another. In both arms, across mental health outcomes and surgical outcomes, the majority of women were considered “recovered” at the post-surgical assessment and remained so at 3-month follow-up (Table 3), albeit with a smaller sample size due to attrition. Overall, recovery was more common for depression and anxiety than PTSD. In the group of women whose surgery was unsuccessful, a higher proportion of intervention participants vs. control participants recovered in depression (80 vs. 66.7%), anxiety (100 vs. 80%), and PTSD symptoms (83.3 vs. 54.5%) by the post survey, although with sample sizes of only 6–11 women.

**Table 3:** Clinical significance analysis of changes in main outcomes of depression^a^, anxiety^b^, and post-traumatic stress^c^ by treatment condition from baseline to post and to 3 months follow-up by success of surgery for women who met the criteria for distress at baseline

	Post				3-month follow-up			
	Recovered^d^	*Reliably improved* ^*e*^	*No change* ^*e*^	*Reliably deteriorated* ^*e*^	Recovered^d^	*Reliably improved* ^*e*^	*No change* ^*e*^	*Reliably deteriorated* ^*e*^
Depression^a^ (*N* = 40)					(*N* = 29)			
*Unsuccessful surgery*								
Control	6/9 (66.7%)	9/9 (100.0%)	0/9 (0.0%)	0/9 (0.0%)	4/5 (80.0%)	4/5 (80.0%)	0/5 (0.0%)	1/5 (20.0%)
Intervention	4/5 (80.0%)	4/5 (80.0%)	1/5 (20.0%)	0/5 (0.0%)	3/3 (100.0%)	3/3 (100.0%)	0/3 (0.0%)	0/3 (0.0%)
*Successful surgery*								
Control	11/12 (91.7%)	11/12 (91.7%)	1/12 (8.3%)	0/12 (0.0%)	9/9 (100.0%)	9/9 (100.0%)	0/9 (0.0%)	0/9 (0.0%)
Intervention	11/14 (78.6%)	11/14 (78.6%)	3/14 (21.4%)	0/14 (0.0%)	12/12 (100.0%)	12/12 (100.0%)	0/12 (0.0%)	0/12 (0.0%)
Anxiety^b^ (*N* = 25)					(*N* = 19)			
*Unsuccessful Surgery*								
Control	4/5 (80.0%)	5/5 (100.0%)	0/5 (0.0%)	0/5 (0.0%)	2/3 (66.7%)	2/3 (66.7%)	1/3 (33.3%)	0/3 (0.0%)
Intervention	4/4 (100.0%)	4/4 (100.0%)	0/4 (0.0%)	0/4 (0.0%)	3/3 (100.0%)	3/3 (100.0%)	0/3 (0.0%)	0/3 (0.0%)
*Successful surgery*								
Control	9/9 (100.0%)	9/9 (100.0%)	0/9 (0.0%)	0/9 (0.0%)	7/7 (100.0%)	7/7 (100.0%)	0/7 (0.0%)	0/7 (0.0%)
Intervention	6/7 (85.7%)	6/7 (85.7%)	1/7 (14.3%)	0/7 (0.0%)	6/6 (100.0%)	6/6 (100.0%)	0/6 (0.0%)	0/6 (0.0%)
PTSD symptoms^c^ (*N* = 44)					(*N* = 32)			
*Unsuccessful surgery*								
Control	6/11 (54.5%)	8/11 (72.7%)	1/11 (9.1%)	2/11 (18.2%)	4/6 (66.7%)	5/6 (83.3%)	1/6 (16.7%)	0/6 (0.0%)
Intervention	5/6 (83.3%)	5/6 (83.3%)	1/6 (16.7%)	0/6 (0.0%)	3/4 (75.0%)	3/4 (75.0%)	1/4 (25.0%)	0/4 (0.0%)
*Successful surgery*								
Control	6/10 (60.0%)	7/10 (70.0%)	2/10 (20.0%)	1/10 (10.0%)	8/8 (100.0%)	8/8 (100.0%)	0/8 (0.0%)	0/8 (0.0%)
Intervention	10/17 (58.8%)	11/17 (64.7%)	5/17 (29.4%)	1/17 (5.9%)	9/14 (64.3%)	10/14 (71.4%)	3/14 (21.4%)	1/14 (7.1%)

^a^Depression measured by Center for Epidemiologic Studies Depression Scale (CES-D), sample includes women who met criteria (CES-D ≥ 16) at baseline (*N* = 40), of which (*N* = 29) returned at 3 months

^b^Anxiety measure by Beck Anxiety Inventory (BAI), sample includes women who met criteria (BAI ≥ 10) at baseline (*N* = 25), of which (*N* = 19) returned at 3 months

^c^PTSD Symptoms measure by PTS Checklist, sample includes women who met criteria (PTS Checklist ≥ 30) at baseline (*N* = 44), of which (*N* = 32) returned at 3 months

^d^Recovered means that change in mental health score meets two criteria: (1) Follow-up score is below the predetermined cut-off point ($\left({\overline{X_f}}^{\ast }{s}_f+{\overline{X_d}}^{\ast }{s}_d\right)/\left({s}_f+{s}_d\right)$ where $\overline{X_f}$ and *s*_*f*_ are the sample mean and sample standard deviation of the functional distribution and $\overline{X_d}$ and *s*_*d*_ are the sample mean and sample standard deviation of the dysfunction distribution; (2) The reliable change index (RCI) score is > − −1.96 where RCI is calculated as $\left({X}_{post}-{X}_{baseline}\right)/\sqrt{2{\left({S}_E\right)}^2}$ where *X*_*post*_ and *X*_*baseline*_ are mental health score at baseline and post and *S*_*E*_ is the standard error of measurement, calculated as ${s}_f\sqrt{1-{r}_{xx}}$, where *r*_*xx*_ is the test retest reliability of the mental health scale used

^e^Reliably improved defined as RCI < −1.96; No change defined as −1.96 ≤ RCI ≤ 1.96; Reliably deteriorated is RCI > 1.96

## Discussion

Women presenting for surgical repair of obstetric fistula had high levels of psychological distress, which underscores the importance of addressing mental health as part of holistic fistula care. In this study, we conducted a pilot trial of a mental health intervention, which was developed through formative research, grounded in psychological theory, and took advantage of the window of opportunity when women were in the hospital for fistula repair surgery [30]. The study demonstrated the feasibility of a clinical trial; participants were successfully recruited from an in-patient fistula ward, screened for study eligibility, enrolled in a longitudinal RCT, and followed over time beyond hospital discharge. The study also demonstrated the feasibility and acceptability of the intervention, which was delivered with high rates of fidelity by a community health nurse and rated positively by participants. Both the intervention and control conditions improved significantly in mental health outcomes following repair. This speaks to the impact of surgical repair, but may also be attributable to the study design, where women were recruited and individually randomized from a single fistula ward, and contamination across conditions was likely common. Given the pilot nature of the study, additional research is needed to rigorously evaluate the impact of the intervention on women’s mental health.

The data presented here clearly highlight the impact of surgical fistula repair on women’s well-being. Long-term psychosocial benefits of surgical repair have been shown in the literature in a number of contexts. Longitudinal studies have demonstrated quantifiable reductions in distress and psychological symptoms following repair [16, 31], and qualitative studies have suggested that following surgery, women with repaired fistulas report improvements in contentment and quality of life [21, 43, 44]. However, there is some indication that mental health recovery may depend on whether or not the fistula repair surgery was successful. Prior observational work in Tanzania and Ethiopia showed that women with continued leaking following repair were more likely to have sustained distress [16, 21, 31]. Our clinical significance analysis provides some evidence to support this hypothesis. It is possible that women with unsuccessful surgeries may particularly benefit from an intervention focused on cognitive reappraisal and coping behaviors, as they continue to deal with the negative impacts of living with a fistula. A larger trial should consider booster sessions and home-based follow-up for women with unsuccessful surgeries, which would promote mental health and coping, and also support women to remain engaged for future surgeries.

Previous studies in Eritrea [39] and Nigeria [45] have examined the impact of counseling and group psychotherapy, respectively, on mental health outcomes for fistula patients receiving surgical repair. While those studies reported significant improvements in psychological functioning, they did not include control conditions, and therefore were unable to determine whether the intervention had an impact above and beyond the surgical repair. Our pilot RCT makes very clear the importance of including a control condition in an evaluation of a mental health intervention for fistula patients.

### Study limitations

As a pilot feasibility study, there are limitations of the study design in size and scope, as well as lessons learned for future research. Given the pilot nature, the study was underpowered to see effects, and both the regression results and the clinical significance analysis should be interpreted with caution and thought of merely as preliminary information to inform a larger study. Since the mental health outcome measures used for regression analysis have different numerical scales, interpretation of the findings should consider that differences observed in estimated means will have different clinical meanings for each scale. While we believe the change in mental health symptoms over time was largely attributable to surgery, there are other possible biases that may have contributed to the lack of preliminary evidence of an observable intervention effect. As an individual-level intervention in a group setting (i.e., a fistula ward), there is a high likelihood of a spill-over between conditions. Additionally, it is likely that all study participants perceived enhanced support through the infrastructure of the intervention study, which included participant incentives, visits and calls to facilitate participant tracking, and assessments that were an opportunity for women to reflect on and speak about some of the hardships they have experienced in their lives. The rapport established with the study staff may have inadvertently biased self-reported mental health symptoms at the follow-up time points, if study participants wanted to please study staff by underreporting mental health symptoms after surgery. The study staff who were involved in data collection were not blinded to intervention condition, due to a limited number of individuals playing multiple study roles. This may have impacted reporting of outcomes and differential follow-up rates, and should be reconsidered for future studies. When considering generalizability of these findings to other settings, both the demographic and obstetric profile of the study participants and the characteristics of the clinical site should be considered.

## Conclusion

Despite clear and consistent evidence of the psychological distress experienced by women living with obstetric fistula, there have been no evidence-based mental health interventions developed for this population. This study attempted a first step towards filling this gap by conducting a pilot RCT of a six-session individual-level intervention that was developed through formative research with local stakeholders and based on psychological theory. The study demonstrated that the intervention was delivered with high levels of fidelity, integrated into clinical care, and received positively by patients. The successful delivery of the intervention by a nurse-level facilitator underscores the possibility of “task-shifting” to non-mental health professionals in this setting, provided there is adequate training and supervision [46]. Future research should evaluate the intervention in a large RCT that is powered to show intervention effects beyond the impact of surgical repair, and to detect potential moderating effects. Given the potential for contamination in an individually randomized trial, a cluster RCT design should be considered. Additional efforts should be made to ensure retention of women over time, especially among women with high levels of distress or whose surgeries were not successful, and differential retention by intervention condition. Finally, given that surgical success may be a moderator of the impact of the intervention, changes to the intervention content and design may be warranted, including flexible intervention content and home-based follow-up for women whose surgery is not successful.

Fistula repair programs provide a life-changing service for women living with an obstetric fistula. These programs should be guided by an ethos of holistic care, addressing not only the clinical condition, but also the accumulated psychological and social effects of living with an obstetric fistula. This will support not only the healing of the patient, but also the successful reintegration of the patient back to her community.
